# The roundtrip to Fairbanks: the circumpolar health movement comes full circle, part II

**DOI:** 10.3402/ijch.v72i0.21608

**Published:** 2013-08-05

**Authors:** Neil J. Murphy

**Affiliations:** Southcentral Foundation, Alaska Native Medical Center, AK, USA

**Keywords:** circumpolar health, history, Congress Proceedings, health research, International Polar Year

## Abstract

**Objectives:**

Evaluate the course of the International Union for Circumpolar Health (IUCH) and the Proceedings of the International Congress(s) on Circumpolar Health (ICCH) in the context of the concomitant historical events. Make recommendations for future circumpolar health research.

**Study design:**

Medline search and historical archive search of ICCH Proceedings.

**Methods:**

Search of all PubMed resources from 1966 concerning the circumpolar health movement. Two University of Alaska, Anchorage Archive Collections were searched: the C. E. Albrecht and Frank Pauls Archive Collections.

**Results:**

Fourteen sets of Proceedings manuscripts and one set of Proceedings Abstracts were evaluated. There was a trend towards consistent use of the existing journals with indexing in Index Medicus; shorter intervals between the Congress and Proceedings manuscript publication; and increased online availability of either the Table of Contents or Proceedings citations.

Recent additions include online publication of full-length manuscripts and 2 instances of full peer-review evaluations of the Proceedings manuscripts. These trends in Proceedings publication are described within the course of significant events in the circumpolar health movement. During this period, the IUCH funds are at an all-time low and show little promise of increasing, unless significant alternative funds strategies are pursued.

**Conclusions:**

The IUCH has matured politically over these years, but some of the same questions persist over the years. There has been a trend towards more rapid dissemination of scientific content, more analytic documentation of epidemiologic study design and trend towards wider dissemination of scientific content through the Internet. Significant progress in each of those areas is still possible and desirable. In the meantime, the IUCH should encourage alternative funding strategies by developing a foundation to support on-going expenses, for example Hildes awards; explore venues to finance Council President and At-Large members travel costs; and seek grants to fund special projects, for example special supplements in the IJCH.

In hosting the 15th International Congress on Circumpolar Health (ICCH), the American Society for Circumpolar Health (ASCH) welcomed the circumpolar health movement back to Fairbanks for the first time since 1967. It had been 45 years since the original Symposium on Circumpolar Health-Related Problems was held in Fairbanks, Alaska. The following is an effort to explore some of the changes in circumpolar health in those many intervening years.

At the outset, let us look at some of the current International Union for Circumpolar Health (IUCH) issues. You may then be able to help the circumpolar health movement decide on how to proceed in its next 45 years. Ironically, these questions have been asked many times during the interim:

- In a rapidly changing Arctic, how can the IUCH best serve the circumpolar health movement?
- Should the IUCH function as a research funding broker?
- Should the IUCH become an individual membership organization?
- and many more ….

## Background

Our understanding of high-latitude processes has increased since the first International Polar Year (IPY) in 1882–1983. In 1957–1958, the International Geophysical Year focused the rest of the planet's attention on the many resources and ambiguities of the Polar Regions. The International Geophysical Year 1957–1958 involved 80,000 scientists from 67 countries, yet human health was not a major area of study. Fifty years later, the circumpolar health community saw the first IPY that included an emphasis on human health. This change in the 2007–2008 IPY research agenda was, in large, the result of the movement of scientists/health workers who have gathered every 3 years since 1967 to improve human health in the circumpolar areas (1). The circumpolar health movement began in the coldest era of the Cold War. The increased interaction of circumpolar scientists and health workers temporally coincided with a process of thawing distrust and conflict.

### The seminal leaders

In the same year as the 1957–1958 International Geophysical Year, the Nordic Council appointed a committee for arctic medical research, which began a process that culminated with the Nordic Council for Arctic Medical Research (NCAMR) Report. The first exploratory human health conference was sponsored by the World Health Organization (WHO) in Geneva from 28 August to 1 September 1962 (2). The WHO Conference on Medicine and Public Health in the Arctic and Antarctic concluded that there was an urgent need to stimulate high-latitude research, especially on health problems. Another impetus that inspired the circumpolar health movement was the Human Adaptability Section of the International Biologic Programme (1964–1974) (3). Fred Milan, a Human Research Physiologist with the Arctic Aeromedical Laboratory for the US Air Force in Fairbanks, coordinated the arctic volume. As a result of these combined processes, the organization of regular symposia on circumpolar health was agreed upon (4).

### The first international symposium

The first person to organize an international circumpolar health symposium was C. Earl Albrecht in Fairbanks, Alaska, in 1967. C. Earl Albrecht, former commissioner of Health for the State of Alaska, envisioned an IUCH for over a decade before the first international meeting became a reality. With the help of the Arctic Institute of North America, the 1967 Symposium on Circumpolar Health-Related Problems had participants from the USSR, Canada, Norway, Denmark, Sweden, Greenland, Iceland and Finland. It was in this symposium that an informal international affiliation was formed. In the 1967 meeting, the decision was made to hold a symposium every 3 years, each in a different country. Twenty years later, these symposia were reorganized to “Congress”(s), to reflect the formation of the IUCH. At the 1967 Fairbanks meeting, an informal organization of the future ASCH was formed. C. Earl Albrecht, with the assistance of Fred Milan and other Alaskan scientists, held this organization together primarily to participate in future international symposia.

### Early international symposia for circumpolar health

There were approximately 100 scientists present at the first symposia held in Fairbanks, Alaska, 23–28 July 1967 (5). The 2nd International Symposium for Circumpolar Health was held from 21 to 24 June 1971 in Oulu, Finland (6). The President of the Symposium was Ole Wasz-Hockert and the chairman of the scientific committee was Henrik Forsius. For almost 2 years leading up to the 2nd Symposium, the entire activities of the NCAMR concentrated on the upcoming symposium (7). The 3rd International Symposium for Circumpolar Health was held from 8 to 11 July 1974 in Yellowknife, Canada (8). The symposium was organized by Otto Schaefer, and Jack Hildes was the Chairman of the Scientific Program. This was the first symposium to feature a specific nutrition section, with nutrition topics also included in nearly every section, for example cardiovascular, blood lipids, and dietary habits of children (9). The 4th Symposium was held in Novosibirsk, USSR, 2–7 October 1978 with V. P. Kaznacheev, Academician of the USSR Academy of Medical Science, Novosibirsk, as Chairman (10). The symposium was sponsored by the Regional Office for Europe of the WHO. There were 330 reports on the current circumpolar health problems. Two volumes of abstracts were published, in English and Russian, for example not full-length manuscripts. In this context, the Finnish–Russian relationships proved to be very useful. During the preparation for the 4th Symposium, Professor Ole Wasz-Hockert made several visits to Russia (11). Denmark hosted the 5th International Symposium for Circumpolar Health from 9 to 13 August 1981 in Copenhagen with over 300 participants formally registered from 17 countries. Bent Harvald was President and Jens Peder Hart Hansen was the General Secretary (12). Scientists came from as far as Argentina and Australia to share Antarctic activities (13). This symposium exposed the practical difficulties, and especially the financial obstacles, in organizing a conference of that size without a financially responsible organization. This was the direct motivation for the formation of a collaborative structure and ultimately resulted in the formation of the IUCH.

### The maturation process: a circumpolar health “movement”

In 1982, C. Earl Albrecht announced progress in the formation of a future IUCH (14). Four adhering bodies had formed for the purpose of guaranteeing quality representation at the International Symposia including the newly formed ASCH and the Canadian Society for Circumpolar Health, with the previously existing Organization of NCAMR and Siberian Branch of the USSR Academy of Medical Science (15).

The 6th International Symposium on Circumpolar Health was hosted by the ASCH and was held in Anchorage, Alaska in 17–21 May 1984 (16). The American Public Health Association eventually produced “The National Arctic Health Science Policy” as a result (17).

### The International Union for Circumpolar Health

The concept of an IUCH was agreed upon at the Copenhagen Symposium in 1981. At the Anchorage symposium in 1984, the main principles were agreed upon and NoSAMF was tasked with finalizing the principles (3). The IUCH's first official organizational meeting was held in Stockholm, Sweden in 18–19 March 1986 (18). In this meeting, the IUCH Constitution was signed and the IUCH established. The interim Board elected at the Constitutional Assembly consisted of Bent Harvald (Denmark), President, Brian Postl (Canada) Vice President, and Ted Mala (USA) Secretary General and Treasurer. As the official meeting of the fledgling International Union for Circumpolar, the next international gathering was entitled a Congress, instead of a Symposium, per se. The 7th ICCH (ICCH) was held in Umea, Sweden, 8–12 June 1987 (19). During the 7th ICCH, the first IUCH General Assembly was convened on 11 June 1987. The General Assembly elected Jens Peder Hart Hansen, the first President of the IUCH. By 24 September 1987 the IUCH had been further organized with Bylaws. The 4 adhering bodies to the new IUCH were joined by a representative from the Scientific Committee for Antarctic Research.

### Recognition of circumpolar movement commitment

The Canadian Society for Circumpolar Health, with the help of a Donner Foundation (20) grant and government support, struck medals in honour of J. A. Hildes, a revered former Canadian health researcher and a mentor to many. Dr. Hildes was originally from Manitoba and died in 1984. The medals were to be given to an outstanding representative from each of the 4 adhering bodies of the IUCH. These are considered the highest award in circumpolar health. The first Hildes medals were awarded in Umea in 1987.

The 8th ICCH was held in Whitehorse, Canada, from 21 to 25 May 1990. Approximately 750 delegates from 20 countries attended (21). In November 1991, the Nordic Society for Arctic Medicine was founded at an inaugural meeting in Stockholm, Sweden. The 9th ICCH was held in Reykjavik, Iceland, from 20 to 25 June 1993. One main topic was the transfer of responsibility for health and health services to indigenous peoples (22). The 10th ICCH was held in Anchorage, Alaska, from 19 to 24 May 1996 and was the first ICCH to utilize the Internet for online dissemination of Congress information (23). At the end of 1996, the Nordic Council of Ministers decided to discontinue funding for the NCAMR. This event signalled the end of the close governmental support for the circumpolar health movement, hence a need for strategies to continue the work of improving the health status in the circumpolar region through partnerships with other funding agencies. The 11th ICCH was held in Harstad, Norway, from 4 to 9 June 2000. The Millennium Congress included Internet access. The 11th ICCH utilized the Internet for modified online and facsimile Congress registration (24).

### Information era speeds up: circumpolar health keeps up

While the use of the Internet for ICCH registration opened up new horizons, the hardbound Proceedings publication process continued to be expensive and time consuming. Despite a dedicated local editorial staff, some Proceedings were not available for 2 years after the Congress. The publication of the 11th ICCH Proceedings began a major shift to more rapid access to the Proceedings material with a goal of publishing within 12 months of the Congress. The 12th ICCH was held in Nuuk, Greenland from 11 to 14 September 2003 with pre-Congress meetings on 8–10 September 2003 was the first to offer complete Internet registration access. The 12th ICCH Proceedings continued the trend towards rapid dissemination of the material by publishing the Proceedings in the International Journal of Circumpolar Health within 12 months of the Congress (25). The publication of the 12th ICCH Proceedings moved the dissemination of circumpolar health information one major step further by publishing the actual Proceedings full articles, both online and hard copy, for example not just an online Table of Contents. This was the first Proceedings that included “Study Design” in the manuscript's Abstract along with Objectives, Methods and Results, and Conclusions. The designation of “Study Design” required the researcher and editor to analyze and document the exact nature of the epidemiologic investigation. At the 12th ICCH in Nuuk, Greenland, Bjerregaard, Young and Curtis provided documentation of a shift of focus from biologic to the sociology of health (26). The 12th ICCH Proceedings were being published just as the *International Journal for Circumpolar Health* initially negotiated to incorporate new academic publishing partners by joining the University of Alaska, Anchorage, University of Oulu and the University of Manitoba. Other academic publishing partners and sponsoring members then joined the IUCH, and the Nordic Society for Arctic Medicine to form the International Association of Circumpolar Health Publishers (IACHP).

### International Association of Circumpolar Health Publishers

The IACHP was established in 2004 to oversee and financially support the *International Journal for Circumpolar Health*. The journal has been in existence since 1972, initially under the name of *Nordic Council for Arctic Medical Research Reports* but later under different more idiomatic names. IACHP consisted of 8 major and some contributing institutions [universities, societies, International Network for Circumpolar Health Research (INCHR)] with a strong commitment to supporting circumpolar health research and in particular the dissemination of research results.

### Mentoring future circumpolar health leaders

The 12th ICCH began the tradition of formally recognizing the talents of emerging scientists, researchers and health workers. In the tradition of the simple, but persuasive mentoring methods that were the hallmark of Jens Peder Hart Hansen, the IUCH Council awarded the Jens Peder Hart Hansen Fellow Award to emerging scientists, researchers and health workers (27).

These awards are funded by a tithe from the ICCH registrants that is deposited in a managed fund to insure future capacity to move the awards to a self-supporting status.

### International Network for Circumpolar Health Research

The International Network for Circumpolar Health Research (INCHR) was established in 2005 as an offshoot of the IUCH Population Health/Surveys Working Group. Following several years of informal discussions among Working Group health researchers, it was decided to more formally approach those activities. In February 2004, at a symposium at the University of Toronto entitled “Populations in Transition: The Health of Circumpolar Indigenous People,” an agreement was signed to form an international organization devoted specifically to circumpolar health research, recognizing the need for continuing networking in the interlude between the international circumpolar health congresses. The activities of INCHR were funded by a 5-year Canadian “Team Grant” that allowed the organization to conduct various activities without levying membership fees.

### Rounding the corner

The 13th International Congress on Circumpolar Health (ICCH 13) was held in Novosibirsk, Russia, from 12 to 16 June 2006. The Congress theme was “The North—The Peace Zone.” ICCH 13 carried on the tradition of real-time web-based dissemination of the conference abstracts. All of the ICCH 13 Proceedings full-length articles were peer reviewed and indexed in the major scientific clearinghouses within 1 year of ICCH 13, both online and in hard copy (28).

The 14th International Congress on Circumpolar Health (ICCH 14) was held in Yellowknife, Canada, from 11 to 16 July 2009. While the conference abstracts were available online in real-time, the organizers chose not to peer review and hence not scientifically index the ICCH 14 Proceedings and instead publish the Proceedings as a Circumpolar Health Supplement with the IJCH 16 months after ICCH 14 (29).

The 15th ICCH 15 was held in Fairbanks, Alaska, from 5 to 10 August 2012. The conference abstracts were available online in real-time, with the Plenary Sessions live streamed on the Internet. The ICCH 15 Proceedings were sponsored by the Arctic Human Health Initiative (AHHI) and are on course to be published within 10 months of ICCH 15 (30). Based on the experience of ICCH 14, the ICCH 15 allowed both a peer track and a non-peer-review track. Both modalities will be available both online and in hard copy, while the peer-review manuscripts will also be indexed in the major scientific clearinghouses.

As of this writing, the 16th International Congress on Circumpolar Health, Focus on Future Health and Wellbeing, is scheduled to take place in Oulu, Finland on 7–13 June 2015.

### The Circumpolar Health Research Network (CirchNet)

At the time of the ICCH 15 in August 2012, the IACHP and the INCHR were in discussions about how to most effectively accomplish their common goals. At the time of this writing, the 2 groups merged to form the Circumpolar Health Research Network (CirchNet) later in 2012 with the coming together of 2 international circumpolar health organizations – the INCHR and the IACHP. This new association aims to:

- promote cooperation and collaboration among health researchers engaged in research in the circumpolar region;
- facilitate the exchange, communication and dissemination of research results and other health data;
- support the training and development of researchers in circumpolar health;
- publish the *International Journal of Circumpolar Health* and other scholarly publications.

### The successes

There has been a trend towards more rapid dissemination of scientific content, more analytic documentation of epidemiologic study design and wider dissemination of scientific content. Key benchmarks include:

#### Indexing of manuscripts

All but two of the Proceedings since ICCH 7 have been indexed in Index Medicus.

#### Peer review

Five of the last 6 Proceedings utilized a full peer-review evaluation, for example ICCH 10, 11, 12, 13 and 15.

#### Online publication

The ICCH 12, 13 and 15 published full-length peer-reviewed Proceedings manuscripts online within 12 months of the Congress. ICCH 14 was published online 16 months after the ICCH. The Health Sciences Information Service of the University of Alaska Anchorage's Consortium Library has obtained copyright permission to scan and archive all the prior ICCH Proceedings online at www.arctichealth.org.

The ICCH13 was the first ICCH to publish the ICCH abstracts electronically on the Internet in real-time, for example published online during the ICCH (31).

#### Scientific method

The ICCH Proceedings included an attempt to delineate manuscript content with structured abstracts including the designation of “Study Design”. The latter requires the researcher and editor to analyze and document the exact nature of the epidemiologic investigation.

#### Economy of scale

Since ICCH 11 and ICCH 12, the local organizing committees have tried to utilize existing resources at the *International Journal of Circumpolar Health* with its “economy of scale” and editorial expertise. These trends will allow local ICCH organizing committees to concentrate on the Congress, Scientific Program, logistics and follow-up. If this trend continues, this also may lead to more uniformity in scientific and editorial practices. On the other hand, at this time the original scientific methods still rely largely on descriptive reviews with rare international collaborative studies, hence the scientific impact factor ratings need to increase to continue to be sustainable.

### The circumpolar health movement completes a full circle

In many respects, the circumpolar movement has completed its first full circle. In 1957, the Nordic Council appointed a committee for arctic medical research, which began a process that culminated with the NCAMR Report. In that same year, the world celebrated the International Geophysical Year. The recent inception of the INCHR will now promote researcher-to-researcher relationships to increase collaboration, support research training at all levels and strengthen the health information system. It is hoped that the INCHR will help improve the scientific methods used by increasing the number of international cohort studies that apply similar methodologies across circumpolar borders. Only 2 such studies were reported in the ICCH 12 Proceedings (32, 33).

The Arctic Council's AHHI added “human health” emphasis to the IPY, just as the circumpolar movement enters a new phase of collaboration. The AHHI advanced the joint research agenda of the Arctic Council (34). The IPY 2007–2008 was an intense, internationally coordinated campaign of research that initiated a new era in polar science. IPY 2007–2008 included research in both Polar Regions and recognized the strong links these regions have with the rest of the globe. AHHI educated and involved the public, and helped train the next generation of engineers, scientists and leaders. The 2010 Arctic Human Health Initiative Circumpolar Health Supplement detailed human health in the Arctic, circumpolar cooperation on Arctic human health, human health and the Arctic Council, the IPY human health proposals, expansion of networks, research proposals, and their outreach education and communication proposals (1).

### Should we spend the IUCH account down to zero?

**Fig. 1:**
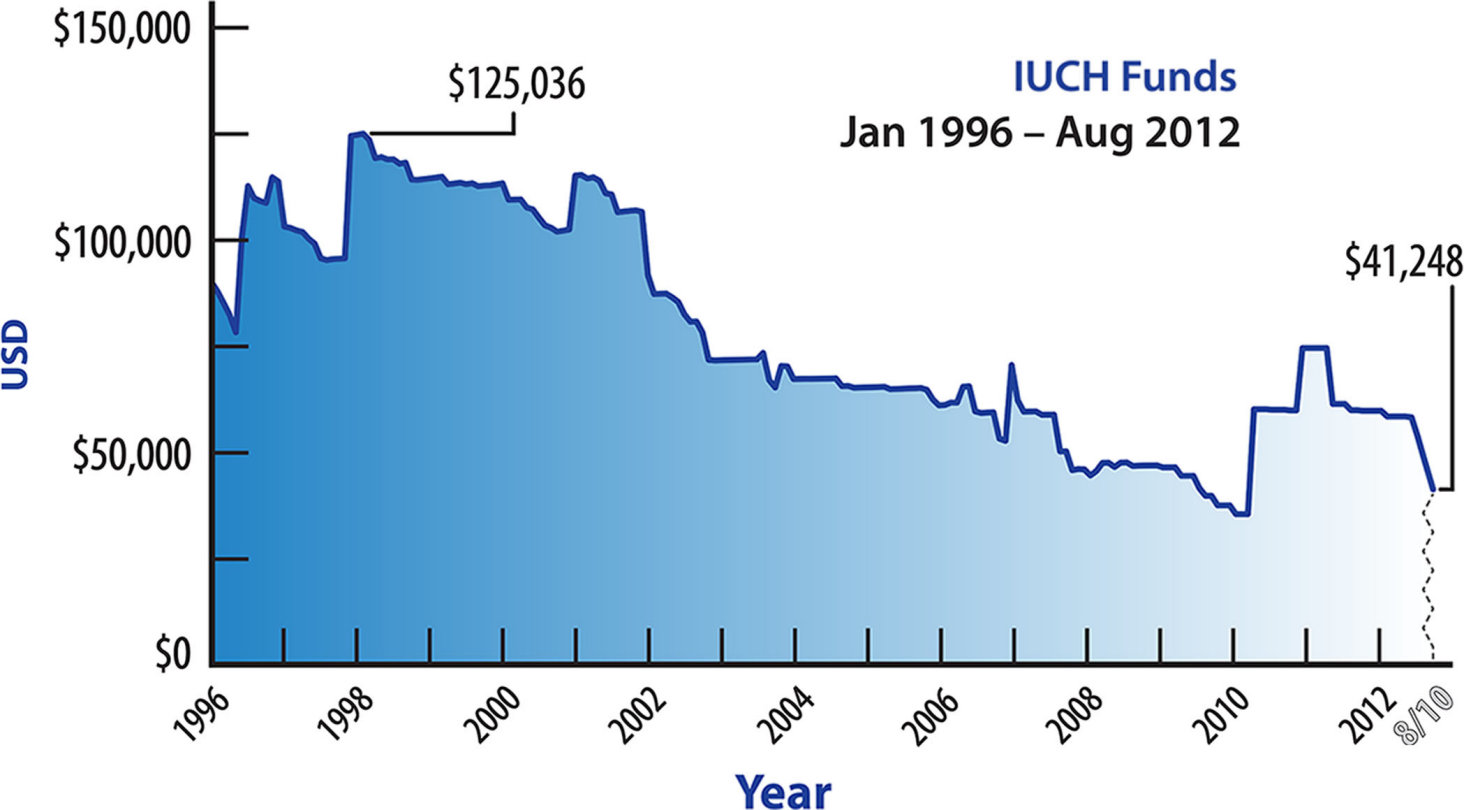
International Union of Circumpolar Health Funds 1996–2010 in USD.

Some IUCH Council members have suggested that the IUCH carry a zero balance in its account by expending all of the funds on special projects and research. Interestingly, this has nearly happened just by inertia alone (Fig. 1).

This question is not merely conjecture because if the steep downward nature of the current slope continues, the IUCH may not have funding to provide for basic operations in the foreseeable future. A zero- balance scenario would occur if an outgoing Council saw the IUCH funds reach a near-zero nadir in conjunction with an ICCH, that is did not leave enough funds for future Hildes awards and the requisite official Council travel expenses for the next ICCH. In that scenario, the funding would lurch from ICCH to ICCH based on individual adhering body's ability to provide start-up capital before ICCH registration fees materialized. A zero-balance system would only favour those adhering bodies with a large membership to capitalize ICCHs. As it is, the current system is more egalitarian, as it allows each adhering body an equal voice in requesting an ICCH in their region.

To better understand the IUCH budget, one must appreciate that there is a cyclic nature to the highs and lows of the IUCH funds. After the governmental support of the European members ended, the ICCHs on the North American continent have been the most heavily subscribed. As the IUCH funding is based on a tithe from the ICCH registrants, the highest account balances occur after the Canadian and American Society's ICCHs due to simple demography. Other IUCH budget factors include: funding of IUCH Council President's travel; funding of At-Large members travel, and special projects. The IUCH budget performs best if: ICCHs are held in geographic venues with easy airline access; the Council President and At-Large members have alternative funding to attend Council meetings or other official activities, and if special projects are funded by outside grants. The IUCH account reached its zenith in 1998 and is nearing its nadir now, despite a large ICCH in Canada in 2009, in part due to the anticipated funding of special supplement if the *International Journal of Circumpolar health* to highlight the work of the IUCH Working Groups (Fig. 1).

The IUCH should explore forming a foundation to provide on-going support of the Hildes Award. Initially, this foundation could be funded through grants and donations, but could also be supplemented with an ICCH tithe system of on-going support, like the Jens Peder Hart Hansen Award. One could easily conjecture that if the original Donner Foundation grant was placed in a similar financial instrument, that the Hildes award would be self-sustaining at this time.

#### Recommendations for the future

- Encourage alternative funding strategies
  1. Develop a foundation to support on-going expenses, for example Hildes awards.
  2. Explore venues to finance Council President and At-Large members travel costs.
  3. Seek grants to fund special projects, for example special supplements in the IJCH.
- Online publication of the ICCH Proceedings within 6 months. This should be possible with careful pre-ICCH planning. With further refinement, the ICCH Proceedings could be published online in decreasing intervals.
- The use of the existing expertise of the *International Journal for Circumpolar Health* should continue and increase.
- Encourage a shift from uni-dimensional descriptive studies to
  1. coordinated circumpolar cohort studies;
  2. interventional studies;
  3. randomized controlled trials.

### What the IUCH is, and what it is not?

- The key strengths of the IUCH are (35):
  1. elected representatives from the national circumpolar societies from all of the Arctic countries;
  2. focused working groups in a variety of fields;
  3. observer status on the Arctic Council;
  4. The ICCH every 3 years;
  5. strong ties to the *International Journal of Circumpolar Health* enabling IUCH Working Group members to publish findings, presented at the ICCH meetings;
  6. information sharing via the IUCH website and the ICCH;
  7. mentoring scholarships and recognition of achievements.
- The IUCH council is currently addressing a number of challenges, including:
  1. limited finances (Fig. 1);
  2. irregular attendance of the IUCH representative at the AC meetings;
  3. lack of regular annual reporting by all WGs to the IUCH;
  4. loss of information/knowledge from past IUCH councils.

### What IUCH is not … yet

One of the persistent issues is whether the IUCH could be a primary funder of circumpolar health research. Given the current financial system whereby the IUCH funds are largely dependent on a tithe based on the number of registered attendees at the ICCHs, the IUCH funds have consistently diminished since the era of government funding (Fig. 1). To that end, the ASCH volunteered to increase their tithe from the ICCH 15 by 50% to improve the IUCH's flagging financial situation.

If the 3 strategies outlined earlier to provide long-term funding are adopted, then the IUCH could explore funding alternatives to serve as a research clearinghouse. The IUCH would be very well positioned to assure that circumpolar health emphasizes multinational collaborations/cohort studies, and that those studies are not duplicative.

Another question that has persistently arisen is “should the IUCH be an individual membership organization?” This type of system would gather dues from individual members, rather than being based on a system of adhering bodies. Demographically at this time, there are proportionally more Canadians in a position to pay such individual dues, compared to other adhering bodies, and they could find their views better represented by numbers alone. However, if the metric was based on the size of the adhering body's base organization, and not its ability to pay individual dues per se, then the Siberian Branch of the Russian Academy of Medical Sciences (AMS-SB) would have the largest number of votes.

In the short term, it may be more expeditious to pursue institutional funding from governmental or non-governmental non-profit agencies, private entities or profit-making ventures. There is IUCH precedent for employing a Secretariat whose main purpose was fundraising, though unfortunately in each of the 2 cases the experiment was not successful. We must better understand that history, so as not to repeat it.

There have also been discussions of re-organizing several of the current circumpolar organizations, for example IUCH, Circumpolar Health Research Network (CirchNet), and so on (36). One initial step may be for the IUCH to welcome CirchNet in as a voting member with 1 vote on the IUCH Council similar to the 1 vote that the Scientific Committee for Antarctic Research (SCAR) is entitled to.

Finally, the IUCH should consider forming a foundation to provide on-going support of the Hildes Award. Initially, this could be funded through grants and donations, but could be supplemented with a tithe system of on-going support, like the Jens Peder Hart Hansen Award, to become self-sufficient.

## Summary

The circumpolar region may now be warming due to a global climate change, but the issues of cold, darkness, isolation, distance, adaptation and permafrost still need study to help human inhabitants adapt. The circumpolar movement began as a vision of a few talented individuals who took the time and energy to pursue international collaboration and cooperation to further increase the knowledge and pursuit of improved circumpolar health. One can hope that the knowledge generated by the circumpolar movement will help stem the onslaught of chronic illnesses associated with socio-economic effects and cultural change. If one takes a broad view of what affects our health as human inhabitants of the circumpolar regions, we are profoundly impacted by both the physical and social environments. Many in the circumpolar health movement believe that those 2 aspects are not mutually exclusive. Indeed, in circumpolar regions, those 2 aspects may be even more interdependent than elsewhere on this planet. We should celebrate the successes that have been accomplished in circumpolar health to date including the more rapid and wider dissemination of scientific content. We should now strive for increasingly rapid dissemination of scientific content, more rigorous scientific methods and increased international collaborative studies. The IUCH should explore alternative funding solutions, for example a foundation to support the Hildes Award; obtain grants for special projects; and create a fund-raising secretariat. The IUCH must continue adapting to change, both political and environmental, while it provides the big umbrella to improve circumpolar health collaboration.
